# Variation in intrinsic resistance of pea aphids to parasitoid wasps: A transcriptomic basis

**DOI:** 10.1371/journal.pone.0242159

**Published:** 2020-11-18

**Authors:** Ailsa H. C. McLean, Benjamin J. Parker

**Affiliations:** 1 Department of Zoology, University of Oxford, Oxford, United Kingdom; 2 Department of Microbiology, University of Tennessee, Knoxville, TN, United States of America; University of California Riverside, UNITED STATES

## Abstract

Evolutionary interactions between parasitoid wasps and insect hosts have been well studied at the organismal level, but little is known about the molecular mechanisms that insects use to resist wasp parasitism. Here we study the interaction between a braconid wasp (*Aphidius ervi*) and its pea aphid host (*Acyrthosiphon pisum*). We first identify variation in resistance to wasp parasitism that can be attributed to aphid genotype. We then use transcriptome sequencing to identify genes in the aphid genome that are differentially expressed at an early stage of parasitism, and we compare these patterns in highly resistant and susceptible aphid host lines. We find that resistant genotypes are upregulating genes involved in carbohydrate metabolism and several key innate immune system genes in response to parasitism, but that this response seems to be weaker in susceptible aphid genotypes. Together, our results provide a first look into the complex molecular mechanisms that underlie aphid resistance to wasp parasitism and contribute to a broader understanding of how resistance mechanisms evolve in natural populations.

## Introduction

Evolutionary interactions with parasites and pathogens contribute to the maintenance of genetic variation in animals [1–3]. Parasites impose strong selective pressures on their hosts, which in turn evolve ways to resist parasitism. Despite the ubiquity of parasites in nature, there is often measurable variation among hosts in their ability to resist parasitism [4, 5]. Factors including costs to defense [6] and the need to defend against multiple different parasite species [7, 8], are thought to maintain variation in resistance within host populations.

Host–parasite evolutionary interactions are predicted to be particularly intense for insect parasitoids, because the successful development of a parasitoid depends on the death of its host. Insect hosts have evolved a suite of molecular mechanisms for resisting parasitoids [9], often involving the innate immune system [10, 11], and natural selection is expected to shape these mechanisms in response to pressure from parasites. However, little is known about the molecular basis of variation in the mechanisms that insects use to recognize and resist parasitoids. The majority of work in this area comes from *Drosophila* species, in which capacity to evolve increased immunity against different common parasitoid threats has been demonstrated by experimental evolution [12]. In these studies, evolution of greater parasitoid resistance is correlated with increasing hemocyte numbers [9, 13]. Identifying molecular mechanisms involved in resistance across a broader variety of host–enemy interactions and studying how these mechanisms vary within host species is critical for understanding how insect immune systems evolve.

One well-studied example of a host–parasitoid interaction is that of aphids and their hymenopteran parasitoids (Braconidae and Aphelinidae). Aphid parasitoids are solitary koinobionts, killing their aphid hosts only at the conclusion of parasitoid development [14]. The aphid genome lacks elements of signaling pathways known to be important in immunity in other insects (including the immunodeficiency signaling pathway, c-type lysozymes, peptidoglycan recognition proteins, and most antimicrobial peptides) indicating that there may be important differences between aphids and other insect groups in the nature of their immune response [15]. Nevertheless, aphids vary in their ability to resist parasitism [16]. It is now recognized that much of this variation in resistance can be ascribed to the presence of facultative symbiotic bacteria [17, 18]. In addition to this symbiont-mediated resistance, pea aphids also deploy their own intrinsic resistance against parasitoids, and there is considerable variation between symbiont-free aphid genotypes in the efficacy of this defense [19]. Aphids therefore combine multiple forms of resistance, but we have little understanding as to how these different immune responses are integrated. Understanding the molecular mechanisms of resistance is an important first step to studying how intrinsic immunity is affected by the presence of protective symbionts.

In this study, we used transcriptome sequencing (RNAseq) to study the molecular responses underlying host-parasitoid interactions between an aphid (*Acyrthosiphon pisum*) and its braconid parasitoid (*Aphidius ervi*). We collected aphids from a single host-plant associated population (‘biotype’) and cured them of any facultative symbionts, and we carried out parasitism assays to identify genotypes that were vulnerable (susceptible) or resistant to parasitoids. We then used RNAseq to measure whole-genome expression of susceptible and resistant genotypes in response to parasitism. First, we ask whether the intrinsic immune response of aphids to the parasitoid is an induced or a constitutive defense: do we see differences in expression between resistant aphids with and without parasitoids? Second, we ask whether vulnerable aphids are mounting an unsuccessful immune response or failing to produce a response at all: are there differences in expression between vulnerable aphids with and without parasitism, and/or between vulnerable and resistant aphids when suffering parasitism? Our findings shed light on molecular mechanisms underlying natural variation in parasitoid resistance that exists among closely-related individuals of the same species.

## Materials and methods

### Experimental insects

Aphids were collected from *Medicago sativa* plants in England between 2003 and 2012 (see Table 1). All lines in this study were initiated from single parthenogenetic females and subsequently maintained in the laboratory feeding on *Vicia faba* (cultivar ‘The Sutton’). Pea aphids reproduce by apomictic parthenogenesis under long photoperiod conditions (16h:8h light:dark cycle) allowing us to maintain genetically-identical stocks in the laboratory for extended periods of time. Microsatellite typing was used to verify that the lines used in experiments were correctly assigned to the ‘*Medicago sativa*’ biotype [20, 21], and to ensure that the lines used were genetically distinct from one another. We screened aphids for the seven known pea aphid facultative symbionts using diagnostic PCR [22], and we then cleared all such symbionts using oral administration of specific antibiotics which do not impact the aphid primary symbiont, *Buchnera aphidicola*, as described in [23]. After antibiotic curing, at least eight generations elapsed before aphids were used in experiments in order to eliminate the possibility of side-effects from the antibiotics. During this period, aphids were regularly screened for their previous facultative symbionts using diagnostic PCR as above, and all aphid lines were confirmed to be symbiont-free eight generations after antibiotic exposure. Prior to use in experiments, we maintained aphids at 14°C in 9cm Petri dishes containing a single leaf of *V*. *faba* inserted into 2% agar; two generations before use in experiments, aphids were moved onto whole two-week-old *V*. *faba* plants at 20°C.

**Table 1:** Information on aphid lines used.

Genotype Name	RNAseq Phenotype	Location Collected	Year Collected	Original Symbionts	Use in previous publications
C218	Susceptible	Eling, UK	2010	Ham, X	[20]
C215	Susceptible	Lincoln, UK	2012	Reg	[20]
C681	Susceptible	Lincoln, UK	2012	Ham	[21]
C308	Resistant	Lincoln, UK	2012	Ham	[20, 24]
C207	Resistant	Lincoln, UK	2012	Ham, X	[20, 24]
C238	Resistant	Beaconsfield	2010	Ham, X	[20]
C689	NA	Milford on Sea	2014	Reg	[25]
C233	NA	Beaconsfield	2010	Ham	[20]
C222	NA	Whitby field	2003	Reg, Ham, Rick	[20]
450	NA	Windsor Ranger’s Gate	2010	None	
C695	NA	Milford on Sea	2014	Ham	[20]
200	NA	Lincoln, UK	2012	None	[20]
C236	NA	Beaconsfield	2010	Ham, X	[20]

Symbiont abbrevations: Ham = *Hamiltonella defensa*, X = X-type (Candidatus *Fukatsuia symbiotica)* Reg = *Regiella insecticola*, Rick = *Rickettsia*.

We maintained parasitoid wasps (*Aphidius ervi*) as inbred stocks in 30cm^3^ cages at 20°C with a 16h:8h light:dark cycle for approximately five years. Wasps were bred on symbiont-free pea aphids (‘stock clone’; from a genotype different to those used in experiments) feeding on *V*. *faba* plants, and periodically provided with a 1:6 honey:water solution for nutrition in the adult stage. Our wasp population takes approximately 10 days to develop from oviposition to pupation. The latter is evident because the aphid becomes a ‘mummy’: a swollen, dried, golden husk which adheres to the substrate.

### Variation in intrinsic resistance to *Aphidius ervi*

Before use in experiments, female wasps were kept in mixed cages (so were assumed to be mated) and given access to aphids of the stock clone to ensure they had oviposition experience. We assessed resistance to *A*. *ervi* in 13 genetically distinct aphid lines. To do this, we placed 15 third instar aphids onto a *V*. *faba* leaf in a 9cm Petri dish, and introduced a young (between 24h and 72h) female wasp into the dish for two hours to allow parasitism (2–8 replicates per aphid line; mean = 7.1). Previous studies have suggested that this is sufficient time to allow most aphids to be attacked but limits self-super-parasitism (laying more than one egg per host) [24]. Aphids were then split into groups of five and transferred to fresh dishes (with subsequent changes every 3–4 days). After 13 days, surviving parasitoids had pupated and aphids were then scored as live or mummified, providing a measure of parasitoid success. We waited until 13 days (two days longer than our standard protocol) to ensure that where we had no mummies observed, this was not the result of delayed mummification for any reason, but genuinely represented no parasitoid survival.

### Exposure to *Aphidius ervi* for transcriptome sequencing

We used the results of the above experiments to choose two groups of contrasting aphids (each comprising three aphid lines): one group was vulnerable to *A*. *ervi* (genotypes C218, C215 and C681) and the other highly resistant (genotypes C308, C207, and C238). We ensured that the lines selected were genetically distinct based on the microsatellite sequencing [20, 21] and did not contain pairs of highly-related genotypes within groups. In order to keep conditions as similar as possible between treatments, we used virgin female wasps for this experiment (as wasps are haplodipoid, this means all eggs laid will be male). Stock aphids were exposed to wasps, and the resultant mummies were collected into size 0 gelatine capsules before hatching. Young virgin female *A*. *ervi* were given access to stock clone aphids 24h before the experiments. The timed-exposure method described above gives a good and repeatable estimate of aphid line vulnerability to parasitoids, but it is not possible to be sure that any individual aphid has received a parasitoid egg or that superparasitism has never occurred. In order to ensure that each aphid contained one parasitoid egg, and so to maximize the chances that any transcriptomic responses to parasitism were detected, here we conducted an observed exposure [26]. We placed aphids in 5cm Petri dishes without leaf material and introduced a female. When we observed an oviposition event, the aphid was removed into a fresh dish using a paintbrush; this continued until 10 aphids of each line had been parasitized. At the end of the exposure period, we placed aphids which had received eggs on to fresh leaves in 9cm Petri dishes in groups of five.

Without prior knowledge of when host-encoded resistance to parasitism might act, we used previous publications on *A*. *ervi* development to select a point at which it seemed likely a response would be mounted, but before that response was likely to have subsided following successful resistance. We chose 96 hours post oviposition, a point at which the larva has emerged from the morula, but before the rupture of the serosal membrane [27, 28]. At 96h post-oviposition, we froze aphids in groups of 10 using liquid nitrogen. Control aphids (not parasitized) were reared and treated identically to experimental aphids but were not exposed to wasps and were also frozen in groups of 10 using liquid nitrogen. Frozen aphids were stored at -80°C until RNA extraction. Note that parasitised aphids differed from control aphids not only in the presence of a parasitoid larva, but also in their having experienced adult female parasitoid wasp presence 96 hours earlier, and in having suffered the small mechanical damage caused by oviposition.

### RNA isolation and transcriptome sequencing

We homogenized frozen aphids with a pestle and extracted RNA using a Trizol/chloroform extraction with isopropanol precipitation and ethanol wash. We degraded genomic DNA with DNase I, and we quantified and quality assessed the RNA using a bioanalyzer (Agilent Technologies 2100 Bioanalyzer System). Libraries were generated using the Illumina TruSeq stranded mRNAseq kit using recommended conditions (including 15 cycles of PCR amplification). Each of the 12 libraries was quality controlled using a bioanalyzer after library prep, and was then multiplexed across 2 lanes of Illumina HiSeq2500 v4 (125 base paired end sequences) yielding a target of 2 x 220 million reads per lane.

### Aphid transcriptome assembly and analysis

We trimmed reads using fastq-mcf (-q 20) to remove low-quality ends and to eliminate any adapter sequences from the raw reads. Trimmed reads were quality assessed using fastqc (https://www.bioinformatics.babraham.ac.uk/projects/fastqc/). We then aligned raw reads using tophat v.2.0.14 [29] implemented in python 2.7.15 to version 2.1b of the pea aphid reference genome [30] (https://bipaa.genouest.org/sp/acyrthosiphon_pisum/), after estimating insert size at 164bp using picard tools v.2.14.1 (http://broadinstitute.github.io/picard). We assigned reads with an unambiguous single alignment to genomic features and obtained read counts using HTseq v.0.6.0, using the “union” overlap mode [31] using a modified version of Official Gene Set v.2.1b, with some duplicated (completely overlapping) gene annotations and unannotated rRNA genes removed from the list of genomic features [32]. We performed a principle components analysis (PCA) using the prcomp function on read counts corrected for library size in R v.3.4.1 to visualize patterns of expression across the 12 libraries.

We analyzed differential expression using edgeR 3.22.3 in R v.3.5.0 [33, 34]. The three resistant genotypes and the three susceptible genotypes were analyzed separately. Genes with low read counts in multiple libraries were removed from the analysis using the filterByExpr function. EdgeR uses empirical Bayes methods to estimate gene-specific biological variation, adjusting read-count values for each gene to compensate for highly-expressed transcripts within each library and for differences in library size across samples. We fit treatment (control or wasp-infected) to each adjusted count-value for each gene in the aphid genome (glmQLFit function), and identified differentially expressed genes using an empirical bayes quasi-likelihood F-test (glmQLFTest function). Genes were interpreted to be significantly differentially expressed at a false discovery rate (FDR) of < 0.1. Last, we used an Enrichment Analysis (Fisher’s Exact Test) of Gene Ontogeny (GO) terms to look for over- or under-represented categories of genes in our expressed gene-set in OmicsBox 1.2.4. The GO index was produced in a previous study [35] using Blast2GO.

### Wasp reads

Six of our RNAseq libraries were from samples of aphids infected with a developing wasp larva. We took two strategies to map these reads. First, we mapped the six libraries generated from aphids infected from wasps to version 3.1 (https://bipaa.genouest.org/sp/aphidius_ervi/) of the recently published *A*. *ervi* reference genome [36]. We used tophat v.2.0.14 implemented in python 2.7.15 for this mapping. Second, we mapped the reads using tophat to a combined reference transcriptome containing transcripts from pea aphids and *A*. *ervi* (using wasp transcriptome v.3.0 and pea aphid reference transcriptome v.2.1b). We used samtools v.1.10 to filter and count the number of read pairs mapped to each aphid or wasp transcript [37].

## Results

**Fig 1:**
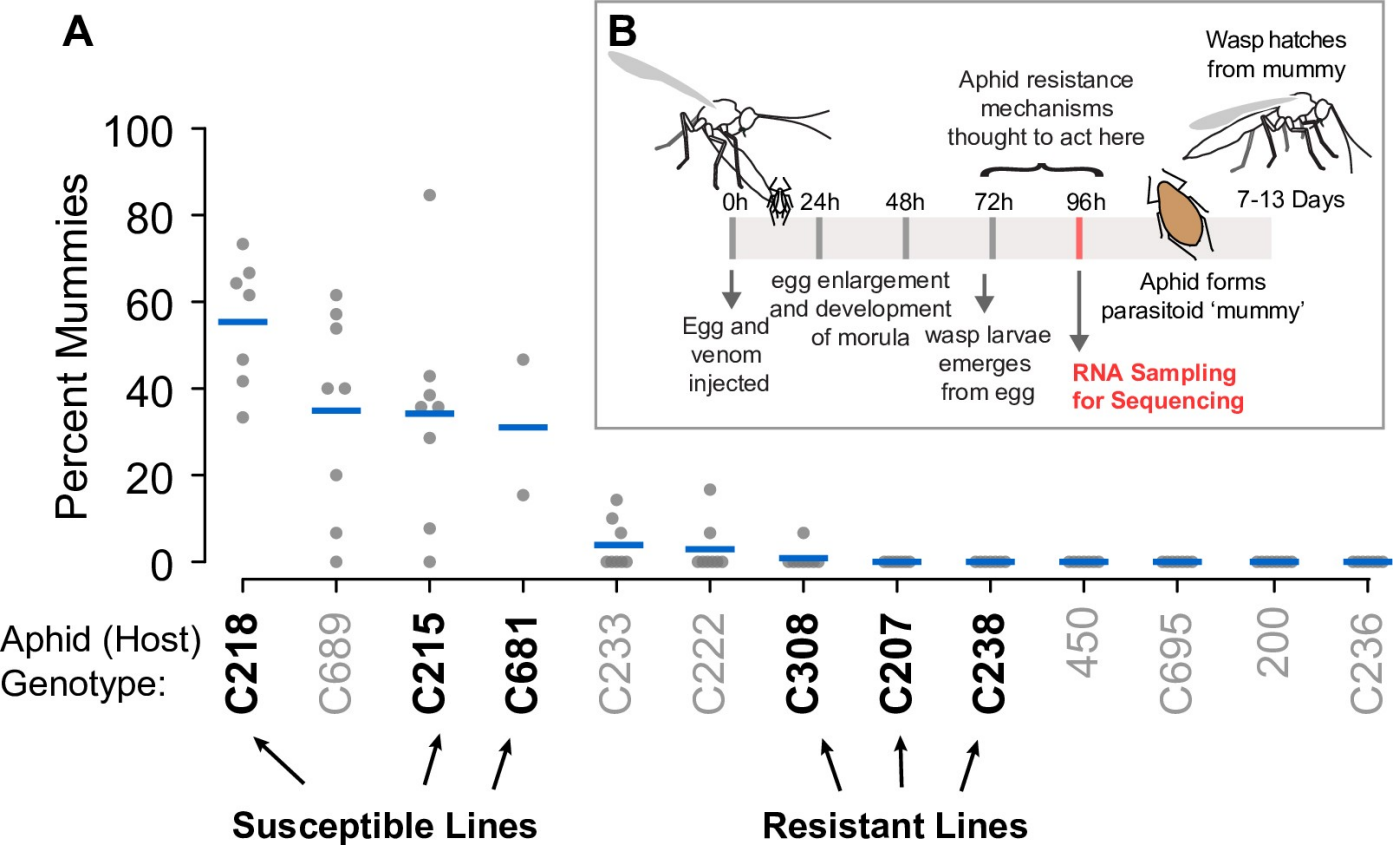
A. Experimental parasitism of aphid genotypes. The y-axis shows the percent of mummies from each dish (replicate). Each replicate is shown with a grey point, with aphid genotypes along the x-axis; the mean parasitism rate for each genotype is shown with a bar. The susceptible and resistant genotypes used in the transcriptome sequencing are highlighted in bold and with arrows along the bottom of the figure. B. Putative life-cycle of aphid-wasp interactions. Aphid resistance mechanisms are thought to act between 72hrs and 96hrs after wasp parasitism.

We found that aphid lines varied in their resistance to *A*. *ervi* (Fig 1A), with lines ranging from apparent total resistance (six lines) to a high proportion of aphids parasitized. From these results we chose three lines with high resistance (lines C308, C207, and C238) and three with low resistance (lines C218, C215 and C681) to be the focus of our transcriptomics study (Fig 1B). We obtained between 28.6 and 52.5 million read-pairs that met quality-control thresholds per library, and on average, 70.8% of reads mapped to the pea aphid reference genome (Table 2). Parasitized and control libraries had similar mapping rates. After removing genes with low read counts in multiple libraries, 12,873 and 12,908 genes were analyzed for differential expression in resistant and susceptible libraries, respectively.

**Table 2:** Sequencing and alignment results.

Library:		Phenotype	Raw Reads (after QC)	Map Rate	Read pairs matched to a gene
Genotype	Treatment				
C215	Control	Susceptible	L: 39,708,146	85.9%	30,154,758
			R: 39,721,089	72.9%	
C215	Wasp	Susceptible	L: 34,999,309	76.0%	24,560,791
			R: 35,001,174	63.1%	
C218	Control	Susceptible	L: 38,636,742	93.0%	33,292,966
			R: 38,635,161	72.4%	
C218	Wasp	Susceptible	L: 51,779,471	90.2%	42,871,591
			R: 51,775,863	66.5%	
C681	Control	Susceptible	L: 36,649,115	64.0%	21,590,879
			R: 36,646,793	45.8%	
C681	Wasp	Susceptible	L: 28,571,997	73.0%	19,323,569
			R: 28,570,673	57.9%	
C207	Control	Resistant	L: 52,491,785	79.9%	38,403,061
			R: 52,484,871	56.4%	
C207	Wasp	Resistant	L: 30,753,767	87.0%	21,196,148
			R: 30,753,761	66.2%	
C238	Control	Resistant	L: 30,222,783	78.8%	21,931,994
			R: 30,220,632	54.5%	
C238	Wasp	Resistant	L: 30,735,751	85.3%	24,147,859
			R: 30,734,847	64.3%	
C308	Control	Resistant	L: 42,543,738	64.4%	25,274,856
			R: 42,540,476	46.8%	
C308	Wasp	Resistant	L: 37,546,282	85.1%	29,569,045
			R: 37,543,589	68.7%	

L: and R: refer to the left and right paired-end reads.

We also mapped reads from the libraries made from wasp-parasitized aphids to the *A*. *ervi* reference genome [38], and to a combined transcriptome made from aphid and wasp transcripts. Both approaches to this analysis showed that our sequencing yielded a low number of wasp reads (Table 3). Wasp read counts were insufficient for an investigation into gene expression, and our results suggest that much more sequencing coverage is needed to analyze wasp gene expression from whole aphids collected at this 96hr timepoint. However, these results confirm the resistance phenotypes of the 6 aphid genotypes used in the transcriptome as measured above: using both mapping approaches, more reads mapped to libraries made from infections using susceptible aphid genotypes than from resistant aphid genotypes (Table 3).

**Table 3:** *A*. *ervi* mapped reads.

Library:	Phenotype	Reads mapped to the *A*. *ervi* reference genome	Wasp read pairs in combined Aphid + Wasp transcriptome
C215 Wasp	Susceptible	131,717 (0.4%) + 133,264 (0.4%)	207,776 (0.5%)
C218 Wasp	Susceptible	762,515 (1.5%) + 771,306 (1.5%)	1,325,091 (1.9%)
C681 Wasp	Susceptible	270,099 (0.9%) + 271,469 (0.9%)	486,838 (1.4%)
C207 Wasp	Resistant	63,988 (0.2%) + 67,956 (0.2%)	224 (0.0%)
C238 Wasp	Resistant	927 (0.0%) + 802 (0.0%)	211 (0.0%)
C308 Wasp	Resistant	25,762 (0.1%) + 26,059 (0.1%)	33,316 (0.0%)

**Fig 2:**
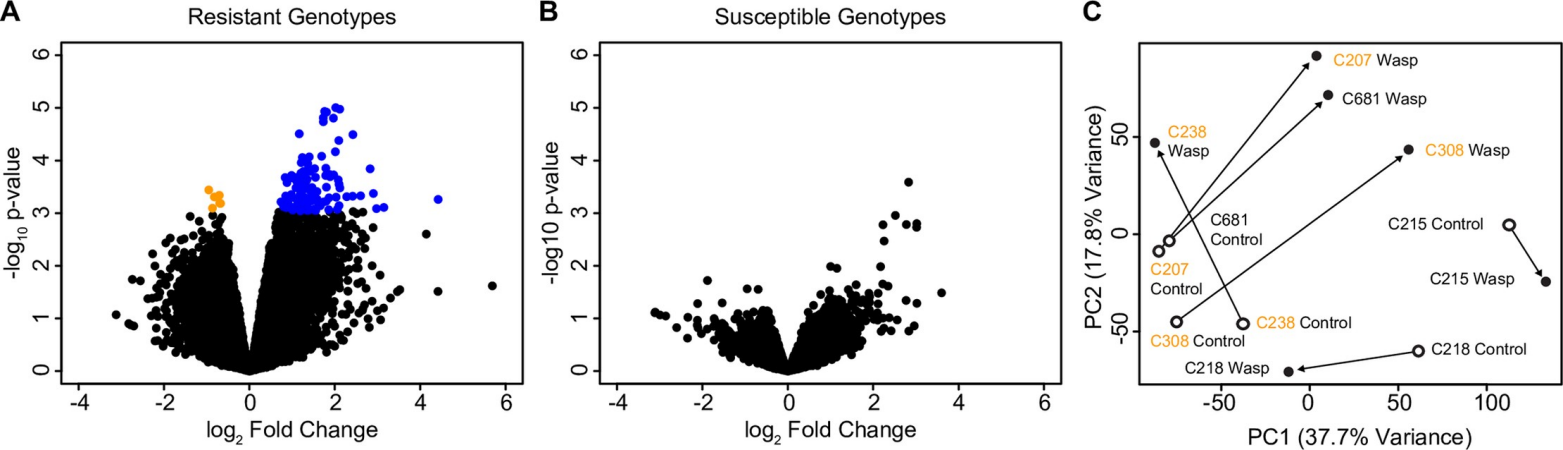
Transcriptome analysis. A&B. Volcano plots of resistant and susceptible genotypes. Each point in the figures represents expression of one gene in the aphid genome. The y-axis of each plot shows the -log_10_ p-value for each gene based on the statistical analysis comparing control vs. wasp-infected aphids. The x-axis of each plot shows the log_2_ fold change of each gene, with genes upregulated in response to wasp parasitism to the right of the figure, and those downregulated to the left. Genes found to be statistically significantly differentially expressed (based on an FDR < 0.1) are highlighted in orange (downregulated) or blue (upregulated). The analyses for resistant and susceptible genotypes were carried out separately and are shown in A and B, respectively. C. Principle Components Analysis (PCA) plot of library size-corrected read counts: PC1 and PC2. This plot is based on the read counts per million of each gene across the 12 libraries. Wasp-infected samples are shown with solid dots, and control libraries with open dots. Resistant genotypes are highlighted in orange. Arrows link the two libraries from each genotype.

Analysis of aphid gene expression showed evidence of differential expression of 104 genes (at an FDR < 0.1) in response to wasp parasitism in resistant aphid lines (Fig 2A). As with all RNAseq studies, our analysis is likely to have missed some genes that were differentially expressed in response to wasp infection and our gene list is not comprehensive. The majority of genes were upregulated in response to parasitism by *A*. *ervi* (99 genes upregulated vs. 5 down-regulated). In contrast, zero genes were differentially expressed at an FDR of < 0.1 among susceptible aphid genotypes in response to parasitism by wasps (Fig 2B). In addition to a lack of significantly differentially expressed genes, the magnitude of differential expression in susceptible genotypes was comparatively low with only 49 genes more than 4X DE (|log_2_ fold change| > 2) compared to 175 in resistant aphids. Overall, patterns of gene expression are suggestive of a stronger reaction to wasp parasitism in resistant compared with susceptible genotypes (Fig 2C and S1 Fig).

Focusing just on the highly-resistant lines, we found that several putative insect immunity genes are upregulated by pea aphids in response to wasp parasitism (S1 Table). These include the gene *hemocytin* (ACYPI003478), which is released by immune cells and plays a role in cell aggregation [39], a *clip-domain serine protease* (ACYPI008297) thought to activate *phenoloxidase* [40–42], and several other proteases and inhibitors (e.g. ACYPI003807, ACYPI004531). These results suggest that mechanisms of cellular immunity are potentially involved in the aphid’s immune response to wasps.

We also found differential expression of a high number of genes involved in metabolism in resistant genotypes. GO analysis of the genes that were differentially expressed by resistant aphids yielded one category of differential gene expression among resistant aphids: “Biological Process” (*carbohydrate metabolic process*—GO:0005975). For example, *glycogen synthase* (ACYPI006932) and *glycogen phosphorylase* (ACYPI001125) were upregulated in resistant aphids in response to wasp infection.

We detected virtually no significant differential expression in our analysis of susceptible aphids infected with wasps. This could suggest that in susceptible aphid genotypes, wasp larvae are successful at hiding from host immune recognition and are not significantly altering their host’s metabolism at this developmental stage. Alternatively, parasitoid venom and compounds released by developing larvae might suppress a response in hosts at this timepoint, implying that the effectiveness of wasp venom varies across host genotypes. We note, however, that there was a high level of variation among susceptible genotypes: Genotype C681, which is phenotypically a susceptible genotype (~40% of exposed aphids became infected with wasps and sequencing libraries revealed a relatively high level of wasp transcripts), qualitatively expressed a pattern of gene expression similar to the three resistant genotypes (Fig 2C and S1 Fig).

## Discussion

The pea aphid is an important model for studies as wide ranging as the molecular basis of phenotypic plasticity, ecological speciation, and host-microbe coevolution [43]. Previous studies have characterized the pea aphid’s immune response to microbial enemies [15, 44], but little is known about how aphids respond to parasitoid wasps—a major source of mortality in natural populations [45, 46]. We have shown first, that pea aphids can produce an induced immune response to the presence of the parasitoid *A*. *ervi*; and second, that the mechanisms underlying aphid intrinsic vulnerability may vary among genotypes.

Aphids have been shown to form melanotic capsules around Sephadex beads, which are used as a proxy for parasitoid eggs [47]. The patterns of expression in response to live wasp parasitism which we uncover in this study suggest that resistant aphids may use these mechanisms of cellular immunity to respond to wasps. For example, we identify that the gene *hemocytin* is upregulated in resistant aphids responding to wasp parasitism. Hemocytin is released by immune cells and thought to play a role in cell aggregation [39]; the increase in expression of this mechanism we identify here could reflect increased proliferation of aphid immune cells [47, 48] or altered activity of these cells. This raises an important caveat of our data, which is that we measured gene expression in whole aphids. The transcriptional response to wasp infection is not expected to be uniform across an entire aphid, and future work would be needed to determine how this response differs in specific cells or tissues, for example in immune cells.

We also found that resistant aphid genotypes differentially expressed genes involved in metabolism in response to wasp parasitism. GO analysis revealed only one category of significantly differentially expressed genes in resistant aphid genotypes: “carbohydrate metabolic process.” We found that genes in this category were upregulated in response to wasp parasitism. Increased expression of these genes may reflect a large-scale metabolic effort on the part of hosts to mount a costly immune response to parasitism [49]; immune costs to resistance have been measured in pea aphids in response to natural enemies in some studies [44, 50] but not others [19], and are likely dependent on ecological context [6, 51].

Of our ‘susceptible’ genotypes, two out of three produced little transcriptomic response to wasp parasitism (Fig 2C and S1 Fig). This is surprising as we predicted that susceptible aphids would experience considerable changes to gene expression [27]. It may be that these changes are highly localized within specific tissues, and so not significant when the whole organism is sampled, or that our 96 hour sampled point fell exactly between the action of maternal venom following oviposition and the beginning of host manipulation by the wasp larva and/or teratocytes. Alternatively, or in addition, the absence of an expression response may reflect an immune evasion or suppression strategy employed by *A*. *ervi* to overcome host defenses, as found in other insect–parasitoid systems [10]. For example, the serosal membrane surrounding the egg and embryo effectively ‘hides’ some parasitoids from the host immune system [52]; alternatively, parasitoid venoms injected at oviposition may act to suppress immune system response [53].

By contrast, one of our three susceptible genotypes (C681) produced a transcriptomic response similar to the resistant genotypes, but nevertheless is relatively vulnerable to parasitism (approximately 40% parasitism in our assays). This finding suggests that even when a transcriptional response is produced it is not necessarily effective. Perhaps there is genotype-by-genotype specificity in the aphid’s intrinsic resistance (as there is for the symbiont-mediated resistance [24]), or perhaps the underlying mechanisms causing genotype C681 to be susceptible to wasps are different from those measured in this study. It is also important to note that our study captured the response at a single time point, and it is possible that immunity might act at different time points in different aphid lines. Moreover, it is almost certain that a transcriptional response will change over time within an aphid individual. The snapshot approach employed here captures one part of the aphid–parasitoid interaction, but further studies using a time-series approach would be valuable in assessing the process of parasitoid infection.

Previous work on pea aphids has demonstrated that a significant proportion of the variability among aphid lines in susceptibility to *A*. *ervi* is independent from the secondary symbiont status of each clone [19]. Our results confirm this finding. By using closely-related aphid lines, all collected from the same host-plant associated biotype, we further show that aphids within a biotype vary in parasitoid resistance. The intrinsically-resistant lines used in this study originally harbored strains of the secondary symbiont *Hamiltonella* that provide specific protection against *A*. *ervi* [24, 54]. Aphids are therefore employing multiple forms of protection against parasitoid wasps simultaneously. Experiments in which protective *Hamiltonella* was artificially introduced into symbiont-free aphids imply that the two forms of protection are redundant, since intrinsically vulnerable aphids are capable of complete resistance in the presence of the symbiont [18, 54]. However, it is possible that maintaining intrinsic resistance is important under specific circumstances when symbiont-mediated resistance might fail, for example, at high temperatures [55].

Our results provide the first steps towards a functional understanding of aphid intrinsic resistance to parasitoids and provide potential targets for functional investigations of the aphid’s response to parasitoids. We also demonstrate that RNAseq provides a viable method for investigating the genetic basis of phenotypic variation between individuals (or as here, asexual lines). In the light of previous studies highlighting the role of symbionts in aphid immunity, future work could explore interactions between innate and symbiotic defenses and the reasons why the two persist alongside one another. More broadly, aphids are important model organisms for understanding community interactions [46, 56]. Knowledge of the molecular mechanisms underlying natural enemy resistance could provide a route to linking observed host–parasitoid interactions in the field to genetic variation in hosts.

## Supporting information

S1 Fig Additional Principle Components Analysis (PCA) plots of library size-corrected read counts: PC3. These plots are based on the read counts per million of each gene across the 12 libraries, showing PC3 with the first two principle components. Wasp-infected samples are shown with solid dots, and control libraries with open dots. Resistant genotypes are highlighted in orange. Arrows link the two libraries from each genotype.
(TIF)

S1 Table A list of the statistically significantly differentially expressed genes in resistant genotypes. (XLSX)

S1 Data Raw data associated with the experimental parasitism of aphid genotypes assay. Aphid genotype is listed under ‘Clone’ in column A. The number of aphids alive at the start of data collection (Live_day_2) and at the end of data collection (Live_day_13) are listed in columns B and C, and the number of parasitoid mummies (Mummy_day_13) is listed in column D. Each row represents a single experimental replicate.
(XLSX)

S1 File A file specifying the commands used throughout the RNAseq analysis. (DOCX)
