# Supplementary figures and images for: Variation in intrinsic resistance of pea aphids to parasitoid wasps: A transcriptomic basis
Source: PLoS One. 2020 Nov 18;15(11):e0242159. doi: 10.1371/journal.pone.0242159 (PMC7673541; doi:10.1371/journal.pone.0242159)

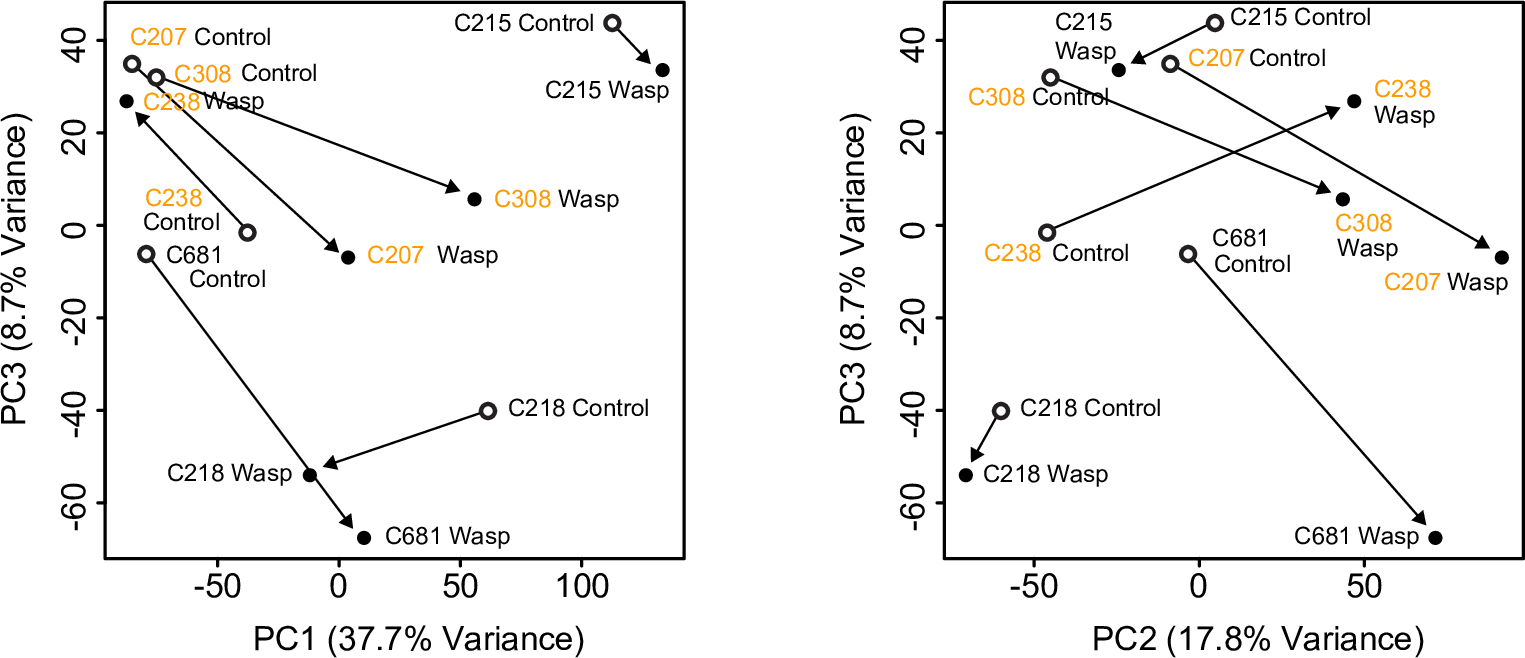

Supplement: S1 Fig — These plots are based on the read counts per million of each gene across the 12 libraries, showing PC3 with the first two principle components. Wasp-infected samples are shown with solid dots, and control libraries with open dots. Resistant genotypes are highlighted in orange. Arrows link the two libraries from each genotype. (TIF) [file pone.0242159.s001.tif]
