# Supplementary material for: Variation in intrinsic resistance of pea aphids to parasitoid wasps: A transcriptomic basis
Source: PLoS One. 2020 Nov 18;15(11):e0242159. doi: 10.1371/journal.pone.0242159 (PMC7673541; doi:10.1371/journal.pone.0242159)
Supplement: S1 File — (DOCX) [file pone.0242159.s004.docx]

**Quality trimming using fastq-mcf**

fastq-mcf RNATruSeq_adapters.fasta raw_left_reads.fastq raw_right_reads.fastq -o left_reads.fastq -o right_reads.fastq -q 20 -p 3

**Tophat v.2.1.0 using Bowtie 2.2.9.0 implemented in Python 2.7.15**

tophat -o output_directory -p 3 -r 164 assembly_scaffolds_INDEX left_reads.fastq right_reads.fastq –-library-type fr-firststrand

**Samtools v.1.10**

samtools sort -n accepted_hits.sam -o accepted_hits_sorted.bam

**Htseq v. 0.6.0**

htseq-count --format=bam --type=exon --idattr="gene_id" --mode="union" --stranded=reverse accepted_hits_sorted.bam genome_features_file > count_file.txt

**EdgeR v.3.22.3 in R v.3.5.0**

#read in data and create a cds object. Generate a sample table based on control/wasp treatment.

#filter out low read count genes:

keep=filterByExpr(cds)

cds=cds[keep, keep.lib.sizes=FALSE]

#Normalize the data by total count.

cds=calcNormFactors(cds)

#Estimate dispersion:

cds = estimateDisp(cds, design=Design_matrix)

#GLM

fit=glmQLFit(cds,Design_matrix)

test=glmQLFTest(fit,coef=2)

tt=topTags(test,n=nrow(cds))

write.csv(tt, file= “output file”)

**Extracting read counts from reads aligned to a reference transcriptome via Samtools v.1.10**

Samtools view -Sb input.bam > filtered.bam

Samtools sort filtered.bam -o filtered_sorted.bam

Samtools index filtered_sorted.bam

Samtools idxstats filtered_sorted.bam > counts.txt
